# A systematic literature review on direct and indirect costs of triple-negative breast cancer

**DOI:** 10.1186/s12962-023-00503-2

**Published:** 2023-11-30

**Authors:** Sadeq Rezaei, Majid Babaei

**Affiliations:** 1 Faculty of Entrepreneurship, University of Tehran, Tehran, Iran; 2 Social Determinants of Health Research Center, Clinical Research Institute, Urmia University of Medical Sciences, Urmia, Iran

**Keywords:** Triple-negative Breast cancer, Economic burden, Direct costs, Indirect costs

## Abstract

**Background:**

Triple-negative breast cancer (TNBC) is an aggressive and therapy-resistant form of breast cancer with a significant economic burden on patients and healthcare systems. Therefore, we completed a systematic review to classify and synthesize the literature on the direct and indirect costs of TNBC.

**Methods:**

Databases including ISI Web of Science, Scopus, PubMed, and Google Scholar were searched for all related articles assessing the economic burden of TNBC from 2010 until December 2022. The quality and eligibility assessments were done accordingly. We adjusted all costs to January 2023 $US.

**Results:**

From 881 records, 15 studies were eligible. We found that studies are widely disparate in the timetable, study design, patient populations, and cost components assessed. The annual per-patient direct costs of metastatic TNBC (mTNBC) were about $24,288 to $316,800. For early TNCB patients (eTNBC) this was about $21,120 to $105,600. Cancer management anticancer therapy costs account for the majority of direct costs. Along with an increase in cancer stage and line of therapy, healthcare costs were increased. Moreover, the indirect costs of patients with mTNBC and eTNBC were about $1060.875 and about $186,535 for each patient respectively.

**Conclusion:**

The results showed that the direct and indirect costs of TNBC, mainly those of mTNBC, were substantial, suggesting attention to medical progress in cancer prognosis and therapy approaches.

**Supplementary Information:**

The online version contains supplementary material available at 10.1186/s12962-023-00503-2.

## Background

Cancer, the most common disease, and the second leading cause of mortality globally refer to several diseases characterized by the progress of unusual cells that grow uncontrollably [1]. Breast cancer (BC) is the fifth foremost reason of cancer-related mortality worldwide [2]. According to statistics, in 2022, after lung cancer, BC is the second main reason of cancer-related death among women globally [3]. The American Cancer Society provides its update for BC where it declares BC alone accounts for 30% of newly diagnosed women in US. In addition, 290, 560 women were diagnosed with BC in 2022. Incidence rates have amplified slightly—by about 0.5% per year on typical—since the mid-2000s [3]. Around 43,250 women will die from BC in 2022 [3]. In the United States, at the beginning of 2022, about 4.1 million women with a history of BC living have been reported. Around 4% of these women are alive with metastatic BC, more than half of whom were initially detected with early-stage (I-III) BC [4]. Overall, in developed countries, the BC incidence is higher than that of in developing countries, however, it has been increasing in developing countries as well [5]. This may be due to a higher prevalence of the known risk factors, lifestyle factors, behavior, late age at any birth, socioeconomic, early age at menarche, low parity, late age at first birth, and late menopause [6–8]. Therefore, genomic alterations are a significant issue that significantly changes the risk profile of BC [9]. BC is a highly heterogeneous cancer that comprises four subtypes including, HER2-positive, triple-negative, luminal A, and luminal B [10, 11]. About 20% of all BC belongs to Triple-negative BC (TNBC) which is most common among women under 40 years of age [10]. TNBC is an aggressive tumor with early relapse and a trend to become in progressive stages [10]. The mortality effects, the economic burden, and social effects of BC are key factors for human society [12, 13]. For example, it was reported that the economic burden for lung cancer, colon/rectal cancer, and BC was $188 billion, $99 billion, and $88 billion in the world, respectively [14]. Consequently, the economic burden of cancers is vital due to the increasing costs of cancer diagnosis and therapy. Compared to patients with non-TNBC, patients with TNBC have advanced cancer stages with a low prognosis. These patients experience a higher hospital resource use and cost of care [15]. As TNBC usually occurs at a younger age, therefore patients with TNBC bear a greater economic burden. Because of the growing incidence rate and extended patient being with the higher management costs of BC care, the economic burden of it has possibly elevated ultimately [16]. The costs of TNBC include direct and indirect costs that mainly driven by hospitalization, emergency department visits, reduction of work productivity or loss of job, and outpatient [17, 18] [19].Patients with TNBC receive common chemotherapies. The effects of novel therapies like immunotherapies on total costs of cancer care are unclear. Furthermore, disability and loss of income, mainly among working-age individuals, increase extra burden to young patients, which known indirect costs [20]. For economic assessment (direct and indirect costs), the approaches used for the quantity of efficiency costs may impact the outcomes of the experiments [21]. Variability in methodology used for the productivity costs could hinder the evaluation of results between different countries. Inconsistency results may arise from the value of local efficiency, patient and type of diseases, social security programs, and epidemiologic situations [22]. Due to the high economic burden (direct and indirect costs) of TNBC, the essential to progress the managing patients with TNBC, especially in developing countries is of excessive standing [23]. Cost of cancer investigations are very useful in defining the cost efficacy of diagnosis and therapies of the cancer and accordingly the optimum use of resources. Patients with TNBC bear several direct and indirect costs. In this study, we aimed to prepare a systematic literature review to summarize the published studies and evaluate the several different types of studies included direct and indirect costs of TNBC.

## Methods

In the present study, we used the Preferred Reporting Items for Systematic Reviews and Meta-Analyses (PRISMA) recommendation to identify, select, and critically evaluate all relevant research published between the years of January 2010 and January 2023 [24].

### Literature search

In this systematic literature review, we searched articles from established literature search electronic databases like, PubMed, ISI Web of Science, Scopus, and Google Scholar from January 2010 and January 2023 (Supplementary table S1). No limitations on publication status were enacted. The keywords used were “Breast cancer”, “economic burden”, " TNBC “, " TNBC burden”, and’’ triple-negative breast neoplasms”. The primary exclusion criteria were: (i) publications that were not peer-reviewed, (ii) publications that lack methodological information, (iii) studies not written in English and (iv) publications that did not primarily focus on BC economic burden. The eligibility criteria for study inclusion are presented in Table 1.

**Table 1:** The eligibility criteria for study inclusion

Criteria	Economic burden studies
Study design	Randomized controlled trials, non-randomized clinical trials, Economic assessments, Observational studies, cross-sectional studies case-control studies
Population	Non-metastatic TNBC, Metastatic, TNBC Early-stage, locally advanced
Outcomes	Direct costs, Indirect costs
Comparators	NR
Interventions	NR

NR: Not restricted

**Table 2:** Relevant studies involved in the systematic review

Title	Paper	Country of study	Population included	Years of diagnosis	Type of study	Study perspective	Setting/ database	Age range	Types of direct costs	Results
The Economic Burden of Recurrence in Triple-Negative Breast Cancer Among Working Age Patients in the United States.	Sieluk et al. (2022) [38]	USA	2340	1999–2017	Retrospective observational cohort study	Payer; patient	OptumHealth Reporting and Insights claims database	Adults 18–65 years	Chemotherapy, hospitalizations	The direct costs were $8575/month higher for metastatic recurrence and $3609/month higher for locoregional recurrence vs. patients without recurrence
Systemic therapy, survival and end-of-life costs for metastatic triple-negative breast cancer: retrospective SEER-Medicare study of women age ≥ 65 years	Sieluk et al. (2021) [23]	USA	302	2010–2016	Retrospective observational cohort study	Payer; patient	SEER Medicare Database	women age ≥ 65 years	Chemotherapy, hospitalizations	Mean per-patient-per-month costs < 30 days before end-of-life/follow-up were $14,100 and $15,600 (2019 USD), respectively
Treatment patterns, risk for hospitalization and mortality in older patients with triple negative breast cancer	Valachis et al. (2021) [25]	Sweden	413	2007–2012	Retrospective observational cohort study	Payer	Cancer database	Women ≥ 70 years old	Chemotherapy	The costs of chemotherapy in older TNBC patients was related to age, cancer stage.
The economic burden of metastatic breast cancer in Spain	De las Heras et al. (2020) [37]	Spain	2923	2010	Observational cohort study	Payer	Simulated incidence-based cohort in Spain	ND	Chemotherapy, hospitalizations	Per patient total costs were $186,535 over 5 years. The economic burden of m mTNBC in Spain is Significant, but differs by HER2 and HR status. HER2−/ HR + patients account for the highest burden due to the prevalence of this category, but HER2+/HR + patients have the highest per patient costs.
Healthcare use and costs in early breast cancer: a patient-level data analysis according to stage and breast cancer subtype	Brandão et al. 2020) [24]	Portugal	703	2012	Prospective observational cohort study	Payer	Cancer center	All	Surgeries, Chemotherapy, Hormone therapy, Targeted therapy, Radiotherapy, Hospitalization	Median cost of care was €9215/patient in stage I. These data provide information for the economic evaluation of innovative treatments for eTNBC and highlight the weight that targeted systemic therapy might have in the overall cost of care among patients with eTNBC.
A population-based comparison of treatment patterns, resource utilization, and costs by cancer stage for Ontario patients with triple-negative breast cancer	Brezden-Masley et al. (2020) [26]	Canada	3271	2012–2016	Retrospective, observational, population based study	Payer	Publicly funded healthcare system in Ontario	All	Outpatient, Home care, Chemotherapy, hospitalizations	Despite a less fre- quent use of all treatment modalities compared to eTNBC. Treatment patterns were aligned with the options available at the time but neoadjuvant treatment rates were low.
Variability in hospital treatment costs: a time-driven activity-based costing approach for early-stage invasive breast cancer patients	Roman et al. (2020) [27]	Belgium	14	ND	Retrospective observational cohort study	Payer	Single breast clinic	age below 40 years	Classical diagnosing—triple assessments, Surgery, Adjuvant chemotherapy, Adjuvant hormonal, therapy, Adjuvant radiation therapy, hormonal therapy,	The average treatment cost for triple negative patients amounted to US$26,923.
Early triple-negative breast cancer in women aged ≥ 65: retrospective study of outcomes, resource use and costs, 2010–2016	Sieluk et al. (2020) [28]	USA	1569	2010–2016	Retrospective observational cohort study	Payer; patient	SEER Medicare Database	patients ≥ 65 years	Outpatient cost, Inpatient cost, Emergent cost	Median overall survival was 23 months/not reached (NR)/78 months, with longer survival at stage II (NR/NR/78 months) than stage III (22/43/38 months). Mean per-patient-per-month costs were $10,620 and $17,872 in neoadjuvant and adjuvant periods. .
Assessing direct costs of treating metastatic triple-negative breast cancer in the USA	Skinner et al. (2020) [29]	USA	608	2010–2016	Retrospective observational cohort study	Payer	Community oncology setting/Vector Oncology Data Warehouse	aged ≥ 18 years	Chemotherapy	The mean monthly cost of first line was $21,908 for 505 treated patients. The majority of costs were attributable to hospitalization and emergency department services.
Overall survival, costs, and healthcare resource use by number of regimens received in elderly patients with newly diagnosed metastatic triple-negative breast cancer	Aly et al. (2019) [30]	USA	625	2004–2011	Retrospective observational cohort study	Payer	SEER Medicare Database	Patients (≥ 66 years)	Physician/clinic, Outpatient, Inpatient,	The mean cumulative (per patient per month) cost per patient was US$73,586 (US$10,084). Mean cost in first and second regimen were US$26,950 and US$33,347.
Treatment patterns, clinical outcomes, health resource utilization, and cost in patients with BRCA-mutated metastatic breast cancer treated in community oncology settings	Houts et al. (2019) [31]	USA	114	2013–2015	Retrospective observational cohort study	Payer	Community oncology setting/Vector Oncology Data Warehouse	Patients (≥ 18 years)	Hospitalization, Emergency room visits, Systemic anti-cancer therapy	Rate of use of infused/parenteral supportive care drugs was 25.5% overall and 36.7% among TNBC patients with 15.8% among HR + patients
Advocacy for a New Oncology Research Paradigm: The Model of Bevacizumab in Triple-Negative Breast Cancer in a French Cohort Study	Mery et al. (2019) [32]	France	45	2011–2018	Retrospective observational cohort study	Payer	Single center/Lucien Neuwirth Cancer Institute	Patients had a mean age of 62 years	Chemotherapy, hospitalizations	A balance needs to be found between healthcare affordability, the high price of progress, and the best medical decision for the patients.
Clinical and economic burden associated with stage III to IV triple-negative breast cancer: A SEER-Medicare historical cohort study in elderly women in the United States	Schwartz et al. (2018) [33]	USA	1244	2011–2013	Retrospective observational cohort study	Payer	SEER Medicare Database	Women who were aged 66 years	Surgery combined with chemotherapy	The mean cost per patient-month (in 2013 US dollars) was $4810 for patients with stage III disease and $9159 for patients with stage IV disease
Patient survival and healthcare utilization costs after diagnosis of triple-negative breast cancer in a United States managed care cancer registry	Başer et al. (2012) [34]	USA	2257	1999–2009	Retrospective observational cohort study	Payer; patient	Managed care setting/IIOM cancer registry	All	Chemotherapy	Annual total healthcare costs, adjusted inpatient costs for patients with eTNBC averaged 77% higher ($8395 vs. $4745, p50.0001). Furthermore, payer reimbursements were higher for TNBC than eTNBC patients ($8213 vs. $4486, p50.0001).
Burden of early-stage triple-negative breast cancer in a US managed care plan	Başer et al. (2012) [35]	USA	1967	1999–2009	Retrospective observational cohort study	Payer; patient	Managed care setting/IIOM cancer registry	All	Office visit costs, Outpatient costs, Pharmacy costs, Emergency room costs	Compared with non-TNBC, early-stage TNBC had significantly higher inpatient costs (all-cause: $9154 vs. $5501; cancer-related: $5632 vs. $2869; for both); and ED costs (all-cause: $303 vs. $182; cancer-related: $240 vs. $138,)

### Data extraction

**Fig. 1:**
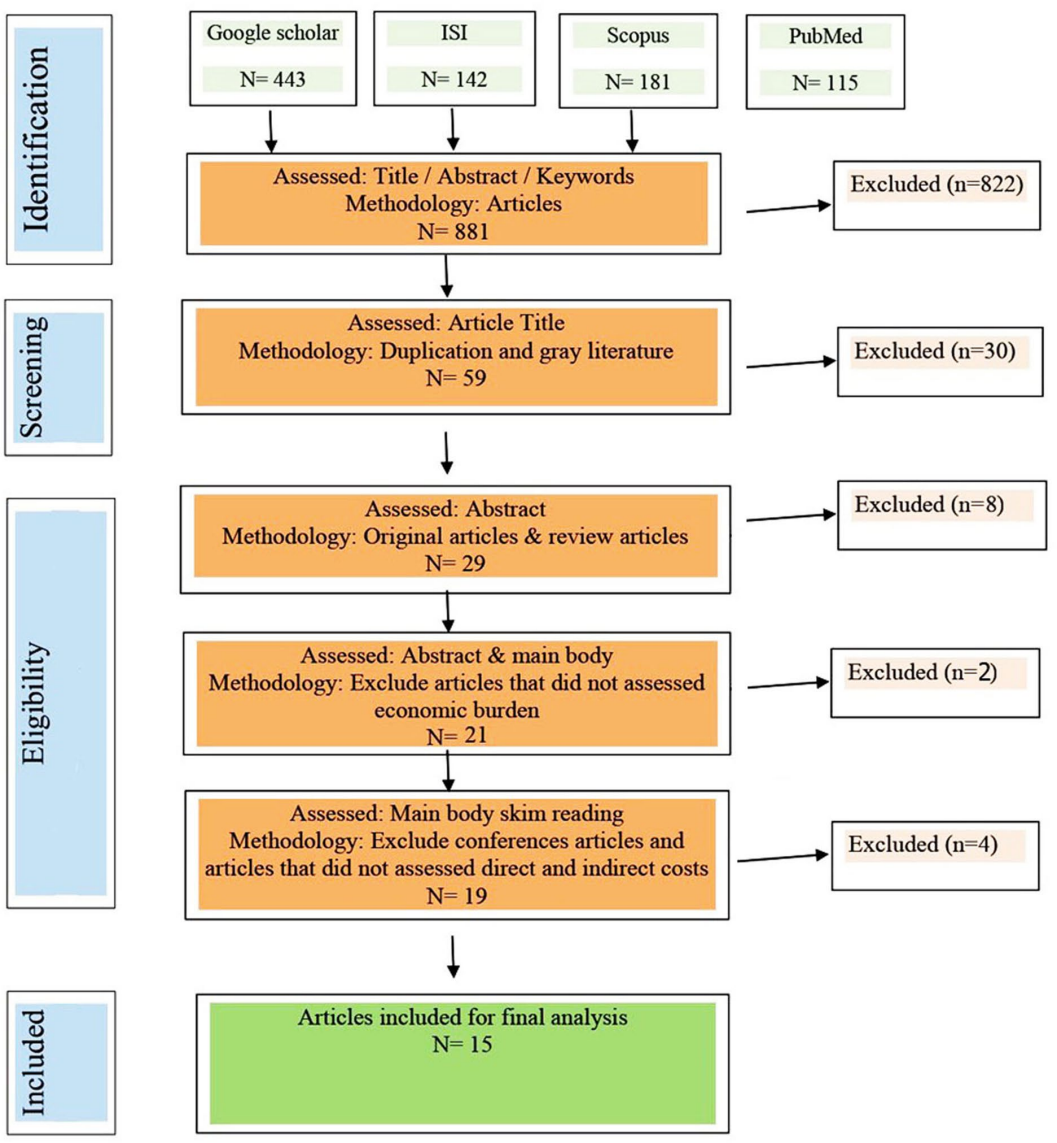
PRISMA flowchart for the systematic literature review on direct and indict costs of triple-negative breast cancer

After a full literature search, a reference manager tool (EndNote) was used to identify and remove potential duplicate articles. Data were extracted from relevant studies, economic evaluations, and clinical trials. For the direct and indirect costs of patients with TNBC, data were extracted from relevant articles. Direct costs are costs that are related to patient care directly, for example: drugs, nursing services, diagnostic imaging, medical supplies, and rehabilitation. Indirect costs - are costs that are not directly attributable to patient care. For instance: reduction of work productivity or loss of job, information technology, general administration, human resources, health records, physical plant and maintenance, and other local facilities. The eligibility criteria inclusion was presented in Fig. 1.

The authors independently reviewed the titles and abstracts of the articles and evaluated full texts for eligibility. Irrelevant studies, duplicated articles, and studies that did not cover the inclusion criteria were discarded. Using a conventional data abstraction process, the authors contributed to preparing the following data from each included article: authors and year of publication, population, setting, country, interventions, age, indirect costs, direct costs, and study design. In the next stage of the selection process, for the risk of bias, extracted data were additionally assessed by Dr Sadeq Rezaie independently [25].

### Quality investigation

The qualitative investigation was passed by two authors (SR and MB) using a checklist. The qualitative investigation was checked by the authors SR. The following points were checked: study design and analysis, economic burdens, scope, type of costs, full text, and availability of the results.

### Cost Adjustment

Because of the diversity in time and place of studies, we adjusted all costs to 2023 (January) USA dollars ($US) for simplifying comparisons between studies as described previously [26]. In articles the cost year was not reported, we considered the publication date as the cost year.

## Results

### Characterization of published and relevant studies

The search and assortment procedure to identify relevant studies, which investigated the economic burden of TNBC, was provided as a flow diagram (Fig. 1). As shown in Fig. 1, we first identified 881 studies related to the economic burden. Then, after a screening process, we found 19 eligible articles for full-text monitoring, finally, 15 articles were analyzed according to the study design criteria in the present review work. Included articles are presented in Table 2.

### Study characteristics

**Fig. 2:**
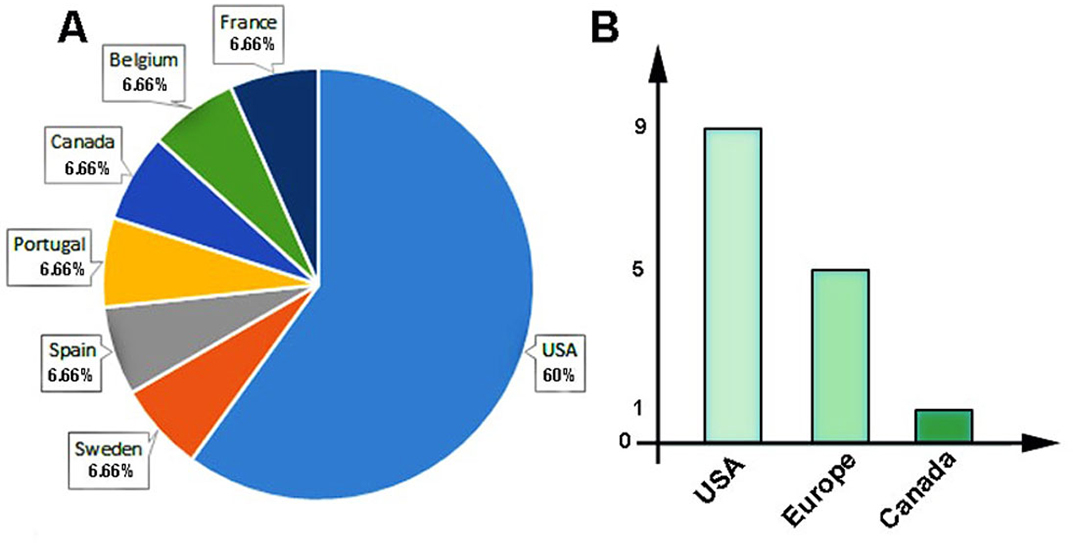
Representative images of study characteristics. Percentage of studies locations **(A)** and the number of studies conducted in different regions **(B)**

The 15 articles published between 2010 and 2022 assessed TNBC-related direct medical costs and indirect costs (Table 2). The direct costs were assessed in 13 studies [27–38]. Two studies estimated both direct and indirect costs [39, 40] (Table 2). As shown in Fig. 2, nine studies were conducted in the USA (60%). The first article was published in 2012, however, almost the studies (n = 7) were done in 2020. In addition, we found that direct costs were the main topic in a total of 15 studies. According to World Bank Classification, all studies were completed in high-income nations (Fig. 2A, B). Five studies investigated the Union’s countries of the European Union. Furthermore, the largest sample size (3271 people) was studied in the article of Brezden-Masley et al. [30], while the smallest sample size (14 people) was studied by Roman et al. [31].

### Direct and indirect costs

For direct costs, in French, the direct costs for patients with mTNBC who first received chemotherapy were ($33,826) [36]. A work conducted by Aly et al. in the USA estimated that direct costs were $87,682 per mTNBC patient [34]. The authors reported that the monthly direct cost of patients with mTNBC who received chemotherapy was lower than that of those who did not receive chemotherapy. In Canada, the annual per-patient costs for patients with mTNBC and eTNBC were $125,049 and $31,284 respectively [30]. For eTNBC, in Belgium, direct costs for per patient were estimated ($29,571) [31]. In Portuguese, it was reported that direct costs for eTNBC were $14,093 per patient after 3 years of follow-up from the diagnosis period [28]. A study by Skinner et al. estimated the highest annual cost (~ $316,800) for treatment of mTNBC [33]. Furthermore, Skinner et al. reported that per month healthcare costs of mTNBC were increased along with lines of therapy [33]. According to Sieluk et al., compared to patients without recurrence, monthly healthcare costs of patients with TNBC with metastatic recurrence and patients with TNBC with locoregional recurrence were $8575 and $3609 higher than respectively. In other word, patients with TNBC with recurrence, mainly metastatic one, had greater direct healthcare costs against patients with TNBC without recurrence [39]. Hospitalization costs of patients with mTNBC who treated anticancer medication were high at the primary medication and low in continuing care but increase in the final care step [37].

For indirect costs, a work showed that the mean indirect costs were $186,535 for each mTNBC patient after 5 years of follow-up in Spain [40] (Table 3). The authors concluded that the economic burden of mTNBC is substantial, but varies by HER2 and HR subtype of BC. HER2−/HR + patients showed the highest-burden because of the prevalence of this tumor, but then HER2+/HR + patients showed the highest costs per patient. Another study in USA reported that monthly indirect costs of patients with TNBC with locoregional relapse were $498.375 against TNBC without relapse; and those with metastatic relapse were $1060.875. Furthermore, patients with TNBC with recurrence showed a 63% higher rate of work loss [39]. De Las Heras et al. reported that costs for overall TNBC population as percentage of total direct and indirect costs comprise 99.85% and 15% over 5 years, suggesting high level of direct costs for patients with TNBC [40].

**Table 3:** Indirect costs of TNBC in two studies

Studies	Authors	Country of study	Type of indirect cost	Type of study	Study perspective	Approach	Cost
The economic burden of metastatic breast cancer in Spain	De las Heras et al. (2020) [37]	Spain	Lost productivity	Observational cohort study	Payer	An incidence-based cost-of-illness model	Monthly per patient ($9.096) per patient over 5 years ($186,535)
The Economic Burden of Recurrence in Triple- Negative Breast Cancer Among Working Age Patients in the United States	Sieluk et al. (2022) [38]	USA	Work loss	Retrospective observational cohort study	Payer; patient	OptumHealth Reporting and Insights claims database	Absenteeism costs were $261 per patient for patients without recurrence, $498.375 for patients with locoregional recurrence, and $1060.875 for patients with meta- static recurrence

## Discussion

In present systematic review, we performed summarized data from 15 published articles on the direct and indirect costs of patients with TNBC. Although we identified other literature reviews on BC or TNBC, however, we have focused on the direct and indirect costs of TNBC in published papers. In our study, we identified there was important heterogeneity across studies that may arise from the timetable, patient population, and cost components assessed. For example, some studies evaluated the direct costs of patients with TNBC aged younger, while some of them evaluated older ones. Analysis of the direct costs represents that there was a substantial association between economic burden and TNBC as well as the severity and stage of TNBC. Studies indicated that patients with TNBC who did not cure antitumor therapy had more diseases with shorter life prospects. Furthermore, per patient, the monthly direct cost was higher in patients with mTNBC who had chemotherapy compared with patients who had [34]. For patients who had anticancer therapy, costs were usually high in the course of primary therapy after diagnosis and lessened significantly in the course of continuing therapy, but amplified again in the final care step [37]. We found that the majority of studies estimated the costs of funding by healthcare payers, however, patient out-of-pocket costs were estimated in four USA studies, which reported $2112 per eTNBC patient and $528 per mTNBC patient per month [27, 32, 38]. In addition, per month costs of patients with mTNBC were augmented with additional lines of cancer therapy [33]. This was additionally confirmed by a work by Aly et al. conducted in the USA that reported that among the chemotherapy received patients, per patient medical direct costs amplified along with the line of therapy [34]. Systemic anticancer therapy comprised around 50% of the whole cost of patients with mTNBC following first-line therapy in the USA, whereas hospitalization was the second main cost [35]. However, the studies that considered longer time prospects for evaluation reported that anticancer therapy comprised only a small part of the total cost of TNBC treatment. It is worth noting that these studies did not consider costs of other payments like caregiver costs or necessary informal care. Indeed, the out-of-pocket costs impose a substantial economic burden on the patients with TNBC who were previously affected by declined work efficiency, cheap income, weakened life quality, and disability. Baser and co-workers in two studies reported that the health strategies of the USA covered the majority of direct costs for patients with eTNBC and mTNBC. This resulted in annual patient costs of about $24,288 and $5280 in eTNBC and mTNBC, respectively [38]. The results revealed the clinical actuality when traditional chemotherapies were the main therapies for TNBC. With the advent of novel tumor therapies, for example immunotherapies treatments for TNBC, the tumor therapy costs will certainly rise. On the other hand, novel therapies may decrease cancer relapse and development, thus lessening annual direct costs per patient. Additional study is necessary to estimate the cost influence and cost efficacy of the new therapies in the life of patients with TNBC. Indirect costs were assessed in an incident cohort study, which was conducted in Spain reporting the economic burden of mTNBC in Spain was substantial but varies by HER2 and HR status of breast tumors over 5 years [40]. In keeping, authors showed that total direct costs comprise 99.85% overall costs. Another study conducted in USA reporting high indirect costs for mTNBC individuals with a high rate of losing job [39]. Patients with mTNBC may lose occupation and cause workplace absenteeism and disability, proposing these were also significant cost constituents from patient and social outlooks. Although these studies revealed indirect costs associated with productivity loss, costs associated with premature death, caregiver, and comorbidity costs were not involved. Therefore, the general indirect costs of TNBC were probably underestimated [41–43]. In this review, most studies included the history of TNBC in diagnosis and therapy, however, it seems that during the pandemic Covid-19, which impact the human societies and governments [44], studies have been done on the alterations during the malignancy and the hamper in the diagnosis and therapy of BC that have also impacted the indirect costs of the disease [45, 46]. Consequently, the characterization of principles and agreement in the methods used to do these studies should be the main concerns for the scientific public [47]. We should note that our review had limitations, for example, we included studies from various regions. Consequently, the evaluations are heterogeneous and the outcomes are not extrapolatable. In addition, we selected studies published in English and omitted non-English articles. We also excluded conference articles. In addition, all costs were adjusted to 2023 $US to simplify comparisons, however, inflation rates and practice designs differ between countries that may affect interpretations.

## Conclusion

The present study represents significant proof of direct costs associated with TNBC that may allow the economic burden of TNBC to be predicted, although cost estimates differ broadly across studies and are fairly challenging to compare. TNBC impose a remarkable economic burden on patients and healthcare systems. The economic burden of patients with TNBC recurrence and progression was substantial with high costs and was increased along with increased cancer severity. Patients with TNBC can be suffered from the indirect economic burden, which is poorly studied in the literature. Further study is essential to evaluate the direct and indirect costs of TNBC therapies to support personalized medicine (e.g., goal-oriented) and medical decisions (e.g., target therapies, participation in randomized controlled trials, etc.) for patients and healthcare payers. These studies may cause an advance in cancer prognosis and therapy methods. In addition, the majority of the studies have been accompanied in high income countries, strategy creators of the healthcare in middle and low income nations need urgencies investigation of such situations.

### Electronic supplementary material

Below is the link to the electronic supplementary material.

Supplementary Material 1

## Data Availability

No data were produced or analyzed in this review study.

## List of Abbreviations

BC: Breast cancer
TNBC: Triple-negative breast cancer
mTNBC: metastatic TNBC
eTNBC: early TNCB
PRISMA: Preferred Reporting Items for Systematic Reviews and Meta-Analyses
