# Supplementary material for: A systematic literature review on direct and indirect costs of triple-negative breast cancer
Source: Cost Eff Resour Alloc. 2023 Nov 30;21:92. doi: 10.1186/s12962-023-00503-2 (PMC10688084; doi:10.1186/s12962-023-00503-2)
Supplement: Supplementary file 1 — Supplementary Material 1 [file 12962_2023_503_MOESM1_ESM.docx]

**A systematic literature review on direct and indirect costs of** **Triple-Negative Breast Cancer**

Sadeq Rezaei ^1^, Majid Babaei ^2^*

1 Faculty of Entrepreneurship, University of Tehran, Tehran, Iran

2 Social Determinants of Health Research Center, Clinical Research Institute, Urmia University of Medical Sciences, Urmia, Iran

***Corresponding author**: Majid Babaei, Assistant Professor, Social Determinants of Health Research Center, Clinical Research Institute, Urmia University of Medical Sciences, Urmia, Iran. [Babaei.m@umsu.ac.ir](mailto:Babaei.m@umsu.ac.ir)

**Supplementary Materials**

**Table S1. the search strategy for the PubMed**

| **Terms** | **Filters** |
| --- | --- |
| Triple-Negative Breast Cancer [TIAB] OR Economic Burden (“Indirect cost”[TIAB] OR “Cost of breast cancer”[MH] OR “Burden of Breast cancer”[TIAB] OR “Triple-Negative Breast Cancer Burden”[TIAB] OR “Economic Burden of Triple-Negative Breast Cancer”[TIAB] OR “Productivity costs”[TIAB] OR “Productivity Lost”[TIAB] OR “Productivity loss”[TIAB] OR “Absenteeism cost”[TIAB] OR "Breast Tumors"[TIAB] OR "Breast Tumor"[TIAB] OR "Breast Carcinoma"[TIAB] OR "Breast Cancer"[TIAB] OR "Malignant Tumor of Breast"[TIAB] OR "Cancer of Breast"[TIAB] OR Medical costs of Breast cancer"[TIAB]) OR “Cost of diagnostic tests"[TIAB] OR “Healthcare costs” "[TIAB]. Hospitalization cost of Triple-Negative Breast Cancer. | Full text, articles, and English language |
